# The role of antibiotic calcium sulfate beads in acute periprosthetic knee infection: a retrospective cohort study

**DOI:** 10.1186/s42836-022-00139-2

**Published:** 2022-09-06

**Authors:** Gianluca Piovan, Luca Farinelli, Daniele Screpis, Stefania Marocco, Leonardo Motta, Giuseppe Palazzolo, Simone Natali, Claudio Zorzi

**Affiliations:** 1 Department of Orthopedics IRCCS Ospedale Sacro Cuore Don Calabria, Negrar di Valpolicella, Italy; 2 Clinical Ortopedics, Department of Clinical and Molecular Sciences, Università Politecnica delle Marche, Ancona, Italy; 3 Department of Infectious-Tropical Diseases and Microbiology, IRCCS Ospedale Sacro Cuore Don Calabria, Negrar di Valpolicella, Italy; 4 Department of Orthopedics and Trauma Surgery, University of Verona, Verona, Italy

**Keywords:** Periprosthetic joint infection, Knee, Absorbable beads, Antibiotic, Implant retention

## Abstract

**Background:**

The study aimed to compare debridement, antibiotics, and implant retention (DAIR) *vs.* debridement antibiotic bead and retention of the implant (DABRI) in terms of infection-free success rate and treatment cost for acute periprosthetic joint infections after total knee arthroplasty (TKA).

**Method:**

Between 2017 and 2020, 32 patients with acute periprosthetic joint infection who were treated by total knee arthroplasty were retrospectively reviewed. The patients were divided into a DAIR group (*n*=15) and a DABRI group (*n*=17). During the DABRI, additional calcium ulphate antibiotic beads were used. Patient age, the Musculoskeletal Infection Society score, microorganisms involved, and success rate were assessed.

**Results:**

The mean age of DAIR group (*n*=15) was 69 years, with 7 being male, and 8 female. The mean follow-up period lasted 30 months. The success rate was 80% (12/15). The mean age of DABRI group (*n*=17) was 64 years, with 10 patients being male and 7 female. The mean follow-up period was 16 months. The success rate was 88% (15/17). There were no significant differences in patient age (*P*>0.05), the Musculoskeletal Infection Society score (*P*>0.05), and success rate (*P*>0.05). A significant difference was found in the follow-up period between the two groups (*P*<0.05).

**Conclusion:**

Both DAIR and DABRI could be used to treat acute periprosthetic joint infections and the outcomes and treatment costs of the two procedures were comparable. Additional use of calcium sulfate beads was safe, but might not improve the treatment result. Randomized controlled studies are warranted for the routine use.

## Introduction

Periprosthetic joint infection (PJI) represents one of the most difficult complications after total knee arthroplasty (TKA) [1]. The incidences of post-TKA PJI reportedly ranged from 0.5 to 2% [2]. Currently, surgical management remains challenging and has a major impact on the patients [3].

According to the Musculoskeletal Infection Society criteria [4], post-TKA PJIs are classified into acute postoperative, acute hematogenous, and late chronic infection. Debridement, antibiotics, and implant retention (DAIR) is an effective therapeutic option for acute PJI [5]. The reported success rates varied from 18 to 100%, depending on the duration of symptoms, time to debridement, type of microorganisms involved, debridement technique used, type of antibiotic, and duration of antibiotic therapy [6–10]. In addition, the use of antibiotic calcium sulfate beads reduces side effects and toxicity caused by systemically administered drugs. Other advantages include a higher local antibiotic concentration, reduced biofilm formation, lowered risk of chronic infection, and protection of implants against bacterial colonization [10–20]. Currently, this debridement antibiotic bead and retention of the implant (DABRI) procedure is an popular treatment for post-TKA PJI [13].

The retrospective study aimed to compare DABRI to DAIR in terms of infection-free success rate and treatment cost for acute post-TKA PJI. The null hypothesis was that the DABRI and DAIR techniques were similar in success rate.

## Materials and methods

Between Jenuary 2017 and December 2020, 41 patients diagnosed with acute post-TKA PJIs were reviewed. Preoperative assessments were conducted by a multidisciplinary team consisting of a microbiologist, an infectious disease specialist, and an orthopedic surgeon. The study was approved by the Institutional review board. Informed consent was obtained from all participants.

**Fig. 1:**
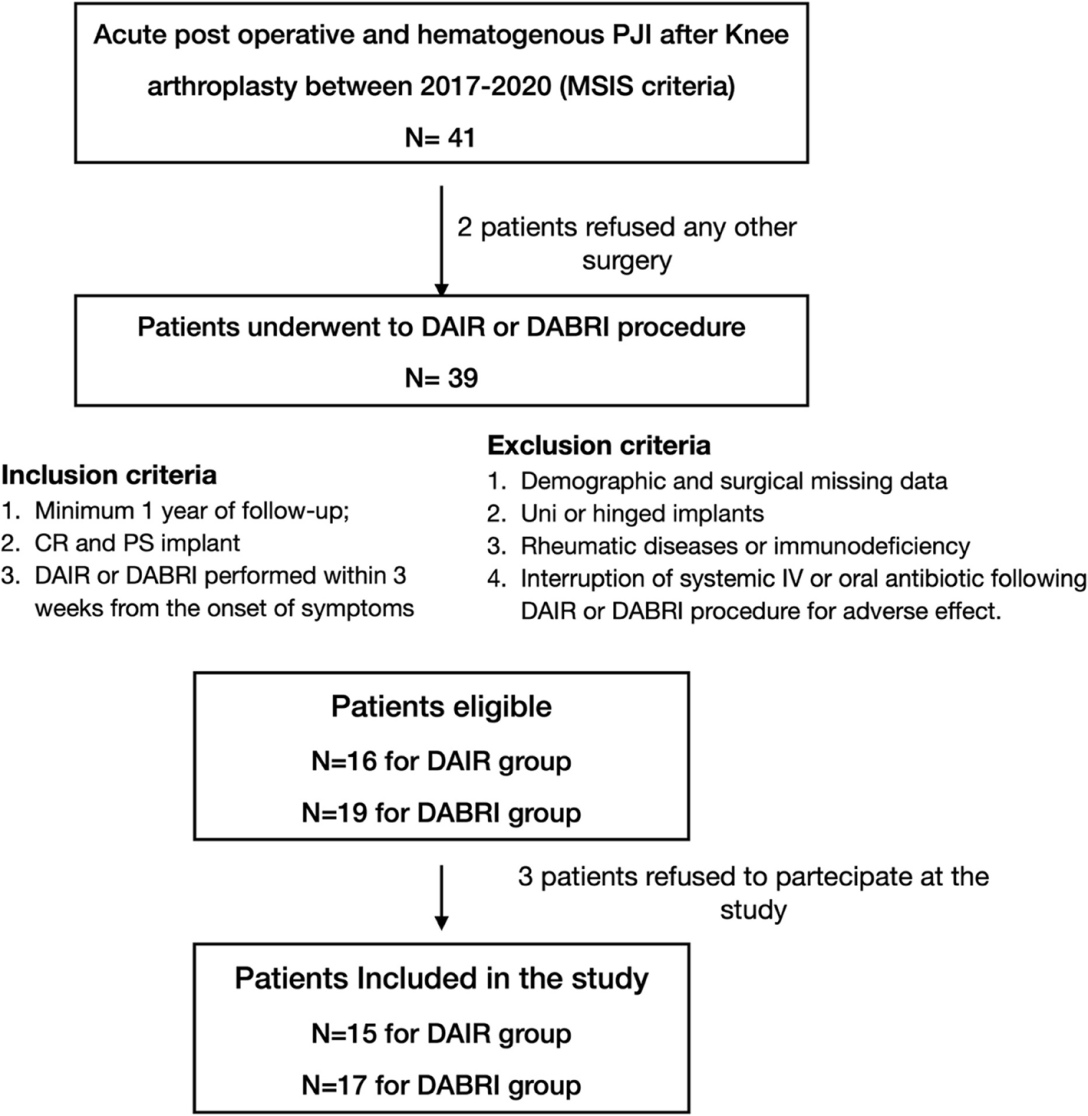
Flowchart of the study. PJI: Prosthetic Joint Infections; MSIS: Musculoskeletal Infection Society; DAIR: Debridement, antibiotics and implant retention; DABRI: Debridement, antibiotics bead and retention implant; CR: cruciate-retaining; PS: posterior-stabilized; Uni: unicompartmental; IV: intravenous

The inclusion criteria included: (1) confirmed diagnoses of acute post-TKA PJIs based on the Musculoskeletal Infection Society criteria [21]. In addition, acute postoperative PJI was defined as infections within 3 months after primary TKA. Acute hematogenous infection was characterized by acute knee symptoms (drainage, pain, joint swelling or effusion, erythema, or sense of warmth) in a previously well-functioning knee after arthroplasty; (2) either a cruciate-retaining or posterior stabilized implant; (3) a patient undergoing either DAIR or DABRI within 3 weeks of symptom onset; and (4) patients were followed up for a minimum of 1 year. The exclusion criteria were (1) the lack of demographics and surgical details; (2) unicompartmental or hinged implants; (3) rheumatic diseases or immunodeficiency; (4) treatment interruption; (5) refusal to receive surgical treatments or to participate in the study (Fig. 1). Finally, 32 patients were included in the study.

Preoperatively, component loosening was assessed based on the medical history, physical examination, knee X-ray, and dual-energy computed tomography. Laboratory tests included analyses of biomarkers in synovial fluid and serum and bacterial culture. All operations were performed in our hospital by the same surgical team (GP, CZ, and DS), with high level of experience (> 15 years of TKA and > 200 knee revision procedures per year). All patients used cemented tibial and femoral components.

### Surgical technique

DAIR procedure was performed under general or regional anesthesia and with tourniquet control. The patient was placed in the supine position. If possible, we made an incision along the previous surgical scar. In patients with multiple surgical scars, the most lateral scar was incised to prevent devascularization of the lateral skin flap [22]. Under sterile conditions, we performed an arthrocentesis to obtain synovial liquid for microbiological and chemical analyses. An aggressive and radical “tumor-like” synovectomy and debridement were performed in the synovial layer and posterior capsule. We collected 5 to 7 samples for microbiological study from the gutters, suprapatellar pouch, and posterior capsule . The modular liner was replaced. The joint cavity was thoroughly irrigated using power-pulsed lavage with saline and dilute povidone-iodine solution [23]. The modular components were replaced in all patients. The wound was closed in layers.

In DABRI procedure, additional alcium sulphate antibiotic beads (Stimulan, Biocomposites Ltd, Kelle, UK) were placed according to manufacturer’s instructions. Antibiotic calcium sulfate beads cost 3000€ for 20 cc of bead volume. The antibiotics added to the calcium sulphate beads were determined by the infection disease consultant based on preoperative findings or empirical data, or both. The resulting paste was then applied, with a spatula, into a silicone bead mold and left to harden. Once fully cured, the beads (10 cc paste volume corresponding to 20 cc bead volume) were placed around knee arthroplasty, in the medial and lateral gutter and in the suprapatellar pouch. No beads were placed in the subcutaneous layer to minimize the risk of wound drainage [24]. Thirteen patients used calcium sulphate powder (10 cc) mixed with vancomycin (1 g) and gentamicin (240 mg) to get 20 cc of bead volume with sterile water. Vancomycin (1 g) and liquid tobramycin (240 mg, 40 mg/ml) were added to 10 cc of powder to get 20 cc of bead volume for 4 patients.

### Postoperative treatment

All patients received a 2-week course of parenteral antibiotics followed by oral antibiotics based on the microbiological findings and as directed by an infectious disease specialist. They were discharged when they were medically fit and suitably rehabilitated and were reviewed two weeks, 4 weeks, 8 weeks, and one year postoperatively, unless closer follow-up was specifically requested. We routinely tested the serum calcium level after DABRI procedure on the first, third, and seventh postoperative days.

### Outcome assessment

Based on the Delphi International Consensus Criteria, PJI was considered eradicated if the infectious symptoms were absent, inflammation markers (C-reactive protein and erythrocyte sedimentation rate) returned to normal, the patient was free from antibiotic therapy, and prosthesis survived for at least 1 year [25]. Patient-related and surgery-related parameters, such as age, body mass index (BMI), comorbidities, American Society of Anesthesiology classification [26], Musculoskeletal Infection Society score [27], types of microorganism involved, were collected. C-reactive protein (CRP) was tested on the 0th, 14th, 30th, 60th, and 90th postoperative days. Success rate was calculated at the final follow-up. Recurrence of PJI caused by the same bacterium was considered a DAIR or DABRI failure.

### Statistical analysis

The data were descriptively and univariately analyzed to look for factors affecting resolution of infection using Chi-square non-parametric tests in the presence of categorical variables and Wilcoxon tests for continuous variables. All effects were considered statistically significant when a *P*<0.05. All analyses were performed using SAS software, version 9.4. After statistical analysis, a *post hoc* analysis was performed using G-Power software (G-Power version 3.1, Düsseldorf, Germany) to assess the power of the study.

## Results

Patient demographics and surgical details are shown in Table 1. Treatment outcomes are given in Table 2.

**Table 1:** Demographics and surgical details of 32 patients

	DAIR group's data (*n*=15)	DABRI group's data (*n*=17)	Statistical data
Age, mean (SD)	69.1 (11.4)	64.2 (8.7)	0.816
Male, *n* (%)	7 (47%)	10 (59%)	0.492
BMI, mean (SD)	30.6 (3.2)	30.9 (3.9)	0.658
Diabetes, *n* (%)	2 (13%)	4 (24%)	0.461
Liver disease, *n* (%)	0 (0%)	2 (12%)	0.401
Cardiovascular disease *n* (%)	2 (13%)	2 (12%)	0.893
Smoking, *n* (%)	1 (7%)	2 (12%)	0.621
Alcohol abuse, *n* (%)	0 (0%)	1 (6%)	0.615
MSIS score	4.5 (2.1)	4.2 (1.8)	0.876
ASA classification	2 (0.5)	2.4 (0.5)	0.723
Failed case, *n*	3	2	0.522
Acute hematogenous, *n*	3	5	0.539
Acute post-operative, *n*	12	12	

*DAIR* debridement, antibiotics, and implant retention, *DABRI* debridement antibiotic pearls and retention of the implant, *n.s.* not significant, *SD* standard deviation, *ASA* American Society of Anesthesiologists classification, *PJI* periprosthetic joint infection, *MSIS* Musculoskeletal Infection Society.

**Table 2:** Clinical results of 32 patients

	DAIR group (*n*=15)	DABRI group (*n*=17)	*P* value
Follow-up (month)			
Mean	30.2	16.1	0.001
SD	13.7	8.1	
Range	12–49	12–37	
Success rate, *n* (%)	12 (80%)	15 (88%)	0.522
Cost based on NHS, €, mean (SD)	19774 (6424)	22363 (9150)	0.923
Length of hospital stay, day, mean (SD)	18.4 (6.0)	21.4 (8.3)	0.712

*SD* standard deviation, *NHS* National Health System (Italy)

The mean age of DAIR group (*n*=15) was 69 years. Among them, 7 were male, and 8 were female. The mean follow-up period lasted for 30 months. The success rate was 80% (12/15). Three patients with acute postoperative (*n*=2) and acute blood-borne infections (*n*=1) failed to respond to the treatment. The staphylococcal microorganisms isolated were *staphylococcus aureus* (2 patients) and *staphylococcus lugdunensis* (1 patient) (Table 3). Three patients had C-reactive protein levels of 98, 110, and 144 mg/dL at the start of treatment. After 90 days, the levels were <5 mg/dL. Postoperatively, systemic intravenous antibiotics were given for 2 weeks, followed by oral antibiotics for a mean of 11.3 weeks (range, 8 to13 weeks). Two patients underwent a two-stage exchange, while one patient received chronic antibiotic suppression but was reluctant to undergo further surgical intervention.

**Table 3:** Two-weeks after systemic antibiotic therapies in DAIR group (*n*=15)

Antibiotics	*n*
Co-trimox + rifampicin	1
Teicoplanin + third-generation cephalosporins + rifampicin	6
Beta-lactam antibiotics	2
Beta-lactam antibiotics + fluoroquinolones	3
Beta-lactam antibiotics +rifampicin	3

Co-trimox, trimethoprim / sulfamethoxazole

The mean age of DABRI group (*n*=17) was 64 years. There were 10 male patients and 7 female patients. The mean follow-up period was 16 months. The success rate was 88% (15/17). The treatment was unsuccessful in 2 patients with acute postoperative infection due to methicillin-sensitive *staphylococcus epidermidis* and methicillin-resistant *staphylococcus epidermidis* isolated from synovial fluid (Table 4). At the start of treatments, C-reactive protein levels in 2 patients were 140 and 112 mg/dL, respectively. The levels were <5 mg/dL after 90 days. Postoperatively, systemic intravenous antibiotics were given for 2 weeks, followed by oral antibiotics for a mean of 11.6 weeks (range, 8 to14 weeks).

**Table 4:** Two-weeks after systemic antibiotic therapies in DABRI group (*n*=17)

Antibiotics	*n*
Teicoplanin / vancomycin + third-generation cephalosporins	9
Teicoplanin / vancomycin + third-generation cephalosporins + rifampicin	6
Beta-lactam antibiotics	2

**Table 5:** C-reactive protein variations in 32 patients

CRP	DAIR (*n*=15)	DABRI (*n*=17)	*P* value
0 day	121.2 (55.9) mg/dL	130.6 (65.4) mg/dL	0.342
14th day	45.2 (39.9) mg/dL	30.67 (18.0) mg/dL	0.418
30th day	19.4 (12.4) mg/dL	11.69 (6.9) mg/dL	0.835
60th day	7.7 (7.3) mg/dL	7.38 (4.1) mg/dL	0.547

*CRP* C-reactive protein; data are shown as mean (standard deviation).

**Table 6:** Microorganisms isolated from 32 patients

Isolated microorganisms	DAIR (***n*** =15)	DABRI (***n***= 17)
Gram-positive		
*Staphylococci*		
CoN *staphylococci*	5	6
Methicillin-sensitive *Staphylococcus aureus* (MSSA)	2	1
Others		
*Streptococcus spp.*	1	2
*Corynebacterium spp*.	1	1
*Propionibacterium acnes*	1	1
Gram-negative		
*Enterobacteriaceae*		
*Escherichia coli*	1	1
*Klebsiella spp*.	0	1
*Proteus spp.*	1	1
Polymicrobical	0	1
Negative Culture	3	2

*CoN* Coagulase-Negative, *spp.* species plural

**Fig. 2:**
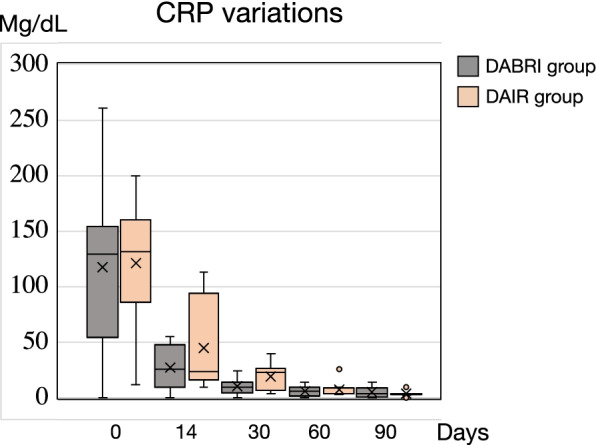
C-reactive protein (CRP) variations over time in DAIR and DABRI groups

We found no significant differences in patient age (*P *= 0.816), sex (*P *= 0.492), Musculoskeletal Infection Society score (*P *= 0.876), and success rate (*P *= 0.522, power = 0.85). We found significant differences in the follow-up period (*P *= 0.001), C-reactive protein reduction (*P *= 0.547 at 60 days) (Table 5 and Fig. 2), and duration of oral antibiotics (*P *= 0.745). Microorganisms isolated are shown in Table 6. No hypercalcemia, heterotopic ossification (HO), or wound drainage was reported.

## Discussion

PJI after TKA is a devastating complication that affects the patient's quality of life. It generates high morbidity and healthcare costs. The diagnosis and treatment of the condition are challenging [28–31]. In accordance with the null hypothesis, we found that the DAIR and DABRI procedures were effective in reducing C-reactive protein levels and yielded similar success rates and incurred comparable treatment costs. Compared to DAIR, DABRI was safer but might not improve the treatment outcomes.

Iza *et al*. [6] treated 22 cases of post-TKA PJIs with DAIR and achieved success in 17 patients. They concluded that hematogenous and *staphylococcus aureus* infections were more difficult to treat than postoperative infections caused by non-*aureus staphylococci* microorganisms. Barros *et al*. [32] treated 38 cases of acute hip and knee PJIs with more aggressive DAIR and attained an overall success rate of 89%. Fierl *et al*. [33] treated 32 patients with acute postoperative and hematogenous knee PJIs using DAIR and reported a failure rate of 48%. They suggested that antibiotic-loaded calcium sulphate beads might not improve the outcomes. However, they did not report the duration of symptoms, time to diagnosis, and time to DAIR, which are associated with biofilm formation that requires single- or two-stage revision procedures to eradicate the infection [17]. Many surgeons agree that the treatments should begin within 3 weeks and even 2 days after symptom onset [8, 9].

S*taphylococcus aureus* may be an independent risk factor for DABRI failure [34–36]. Calanna *et al*. [13] used DABRI for the first time for the treatment of acute knee PJIs. Two patients failed to respond due to methicillin-resistant *Staphylococcus aureus* infection, even though the level of C-reactive protein after 30 days of treatment decreased in both cases [37–39].

Complications of DABRI include wound drainage, hypercalcemia, and heterotopic ossification. The use of a high volume of beads (> 20 cc) and subcutaneous placement may result in wound drainage [24]. A high volume of beads may also cause transient hypercalcemia, especially in patients with chronic osteomyelitis, open fractures, and combat injuries [40]. In addition, patients with renal insufficiency, calciopathy, and parathyroid diseases should be treated with additional caution to prevent hypercalcemia [41]. Serum calcium and creatinine levels should be monitored within 24 to 48 hours after surgery. The cause of heterotopic ossification remains unknown but may involve the use of calcium beads, skeletal muscle injuries, and intraoperative periosteal stripping [42, 43]. Calcium sulfate beads may increase wear in a prosthetic joint, but preliminary studies found that this treatment might not increase polyethylene wear [44].

This study has limitations. First, a retrospective design might introduce selection bias. Randomized controlled trials should be conducted to investigate the efficiency of calcium sulfate beads in relation to different variables, especially bacterial species. Second, the sample size was too small and might not be truly representative of the patients. Third, all patients were operated by the same surgical team and assessed by the same infectious disease specialist. Their experience improved over time, and their preference and ability might have influence on treatment outcomes.

## Conclusion

Both DAIR and DABRI can be used to treat acute post-TKA PJIs and yield similar outcomes and treatment costs. Additional use of calcium sulfate beads is safe but may not improve the treatment. Randomized controlled studies are needed for definitive recommendation of its routine use.

## Data Availability

All data generated or analyzed during this study are included in this published article.
